# Triple network in adolescents with borderline personality disorder, early traumatic experiences and dissociative symptoms: An eloreta study

**DOI:** 10.1192/j.eurpsy.2021.384

**Published:** 2021-08-13

**Authors:** C. Di Maggio, C. Massullo, C. Imperatori, O. Palazzolo, B. Farina, M. Brinciotti, M. Ferrara, V. Guidetti, A. Terrinoni

**Affiliations:** 1 Department Of Human Neuroscience, Section Of Child And Adolescent Neuropsychiatry, Phd Program In Behavioral Neuroscience, Sapienza University of Rome, RM, Italy; 2 Department Of Human Science, Cognitive And Clinical Psychology Lab, European University of Rome, Rome, Italy; 3 Department Of Human Neuroscience, Section Of Child And Adolescent Neuropsychiatry, Sapienza University of Rome, Rome, Italy

**Keywords:** Borderline personality disorder, triple network, adolescents, eeg functional connectivity

## Abstract

**Introduction:**

Triple Network Model (TNM), which considers the dynamic interaction between Default Mode (DMN), Salience (SN), and Central Executive (CEN) networks, explains clinical features in mental disorders from a neurophysiological perspective. Some studies highlight the increased connectivity in TNM in adults with Borderline Personality Disorder (BPD), but little is known about adolescents.

**Objectives:**

The aim of our preliminary study was to investigate TN functional connectivity (FC) in BPD adolescents with a history of traumatic experiences, and its correlation with dissociative symptoms.

**Methods:**

15 BPD adolescents (DSM-5 criteria) with early traumatic experiences were compared to 15 healthy controls, matched for sex and age. Dissociation Questionnaire (DIS-Q) was administered. Eyes-closed resting-state (RS) EEG recordings were performed (19 electrodes; 10- 20 system) and analyzed using Exact Low-Resolution Electromagnetic Tomography software (eLORETA). FC was computed for all frequency bands and 9 Regions of Interest for TNM.

**Results:**

BPD adolescents showed a hyper-connection between CEN and DMN [dorso-lateral prefrontal cortex (dlPFC) and posterior cingulate cortex (PCC); PCC and left posterior parietal cortex (PPC)] and within the CEN (left and right PPC). The strength of PCC-dlPFC and left-right PPC connections was correlated with dissociative symptoms severity.

**Conclusions:**

FC alterations can already be identified in BPD adolescents, supporting the need for early diagnosis. Normally DMN and CEN show opposite functioning. In our BPD adolescents, the absence of this “anti-correlation” reflects the typical confusion between internal and external mental states, which clarify their difficulties in metacognition or mentalization. Moreover, in dissociative symptoms, two CEN nodes are also involved, not only DMN as previously described.

**Disclosure:**

No significant relationships.

